# Associations of Variation in Retinal Thickness With Visual Acuity and Anatomic Outcomes in Eyes With Neovascular Age-Related Macular Degeneration Lesions Treated With Anti–Vascular Endothelial Growth Factor Agents

**DOI:** 10.1001/jamaophthalmol.2020.3001

**Published:** 2020-08-20

**Authors:** Rebecca N. Evans, Barnaby C. Reeves, Maureen G. Maguire, Daniel F. Martin, Alyson Muldrew, Tunde Peto, Chris Rogers, Usha Chakravarthy

**Affiliations:** 1 Clinical Trials and Evaluation Unit, Bristol Trials Centre, Bristol Medical School, University of Bristol, Bristol, United Kingdom; 2 Department of Ophthalmology, University of Pennsylvania, Philadelphia; 3 Cole Eye Institute, Cleveland Clinic, Cleveland, Ohio; 4 Queen’s University of Belfast, Royal Victoria Hospital, Belfast, Ireland

## Abstract

**Importance:**

When initiating anti–vascular endothelial growth factor (VEGF) treatment for patients with neovascular age-related macular degeneration (nAMD), knowledge of prognostic factors is important for advising patients and guiding treatment. We hypothesized that eyes with greater fluctuation in retinal thickness over time have worse outcomes than eyes with less variation.

**Objective:**

To investigate whether visual and anatomic outcomes in eyes with nAMD initiating anti-VEGF treatment are associated with fluctuations in retinal thickness.

**Design, Setting, and Participants:**

In this study using data from the Comparison of Age-Related Macular Degeneration Treatments Trials (CATT) and the Inhibition of VEGF in Age-Related Choroidal Neovascularization (IVAN) randomized clinical trial, people with previously untreated nAMD were included. Data were collected from February 2008 to November 2012, and data were analyzed from April 2017 to April 2020.

**Main Outcomes and Measures:**

Foveal center point thicknesses (FCPTs) were extracted from 1165 study eyes from CATT and 566 study eyes from the IVAN trial, excluding those with 3 measurements or less. For each eye, the SD of FCPT was calculated. Eyes were grouped by FCPT SD quartile. Associations of FCPT SD quartile with outcomes were quantified at month 24 or the last available visit by linear or logistic regression, adjusting for baseline best-corrected visual acuity (BCVA) and randomized allocations to drug and treatment regimen, for BCVA, development of fibrosis, and development of macular atrophy.

**Results:**

Of the 1731 included patients, 1058 (61.1%) were female, and the mean (SD) age was 78.6 (7.4) years. The median (interquartile range) FCPT SD was 40.2 (27.1-61.2) in the IVAN cohort and 59.0 (38.3-89.4) in the CATT cohort. After adjustment for baseline BCVA and trial allocations, BCVA worsened significantly across the quartiles of FCPT SD; the difference between the first and fourth quartiles was −6.27 Early Treatment Diabetic Retinopathy Study letters (95% CI, −8.45 to −4.09). The risk of developing fibrosis and macular atrophy also increased across FCPT SD quartiles. Odds ratios ranged from 1.40 (95% CI, 1.03 to 1.91) for quartile 2 to 1.95 (95% CI, 1.42 to 2.68) for quartile 4 for fibrosis and from 1.32 (95% CI, 0.90 to 1.92) for quartile 2 to 2.10 (95% CI, 1.45 to 3.05) for quartile 4 for macular atrophy.

**Conclusions and Relevance:**

Greater variation in retinal thickness in eyes with nAMD during treatment with anti-VEGF was associated with worse BCVA and development of fibrosis and macular atrophy in these post hoc analyses, despite protocol-directed treatment frequency. Practitioners may want to consider variation in retinal thickness when advising patients about their prognosis.

## Key Points

**Question:**

Are fluctuations in retinal thickness associated with visual and anatomic outcomes in eyes with neovascular age-related macular degeneration treated with anti–vascular endothelial growth factor drugs?

**Findings:**

In this study of 1731 participants from 2 randomized clinical trials, increasing variation in retinal thickness was associated with worse outcomes in post hoc analyses of protocol-directed treatment regimens.

**Meaning:**

These findings suggest that fluctuating activity may be a marker for poor prognosis in eyes with neovascular age-related macular degeneration treated with anti–vascular endothelial growth factor drugs.

## Teaser

This study investigates whether visual and anatomic outcomes in eyes with neovascular age-related macular degeneration (AMD) initiating anti–vascular endothelial growth factor (VEGF) treatment are associated with fluctuations in retinal thickness.

## Introduction

Treatment of neovascular age-related macular degeneration (nAMD) has been transformed by intraocular injection of therapies that inhibit vascular endothelial growth factor (VEGF).^1^ The goal of therapy is to achieve a macula free of exudation.^2^ Clinicians use optical coherence tomography (OCT) criteria (indicating disease activity) to tailor retreatment.^3^ Although it is critically important to optimize the treatment regimen to achieve the best possible outcome, there is also a desire to achieve this goal with the fewest treatments and patient visits.^4^ However, after initial control of active disease has been achieved, recurrence of fluid does not appear to have a clinically important adverse effect on functional outcome if managed with prompt retreatment.^5,6,7^

Two large contemporaneous randomized clinical trials, the Comparison of Age-Related Macular Degeneration Treatments Trials (CATT)^8,9^ and the Inhibition of VEGF in Age-Related Choroidal Neovascularization (IVAN) trial,^10,11^ randomized participants to 2 drugs, ie, ranibizumab and bevacizumab, and 2 treatment regimens, ie, monthly treatment or monthly review with treatment withheld if study eye lesions were quiescent.^8,9,10,11^ When the difference in visual outcome between treatment with ranibizumab or bevacizumab was evaluated within dosing regimens, no clinically important difference was detected at either 1 or 2 years after randomization in either trial,^8,9,10,11^ consistent with results from other clinical trials comparing the 2 drugs.^12^ Studies in clinical practice have shown, on average, recovery of visual acuity providing that treatment is administered promptly when retinal thickness increases (a marker for recurrence of lesion activity).^13^

It is challenging to distinguish the effects of variation in retinal thickness from the effects of wide variation in treatment-related responses between individuals that may arise from nAMD lesion type, size, and activity.^3^ The timing of retreatment is also influenced by other factors, such as missed visits and clinician availability. We elected to study the association of eye-level SD of retinal thickness with outcomes in post hoc analyses of data from the CATT and IVAN trials^8,9,10,11^ to minimize the influence of the latter factors. These trials achieved excellent adherence to monthly follow-up at which disease activity was reviewed and treatment restarted if necessary. The 2 trials used similar methods of data capture, allowing individual participant data to be combined.

## Methods

The CATT trial^8,9^ randomly assigned participants with newly diagnosed nAMD to 4 treatment groups: bevacizumab or ranibizumab, either given monthly or when required (pro re nata [PRN]); the PRN regimen did not specify an initial number of injections. Participants were observed for 2 years. At 1 year, participants in the monthly treatment groups were rerandomized to monthly or PRN treatment. The IVAN factorial trial^10,11^ also compared bevacizumab vs ranibizumab and compared monthly vs PRN regimens in previously untreated eyes with nAMD; the PRN regimen mandated a cycle of 3 monthly injections when treatment was restarted after a period of lesion inactivity. Both trials tested noninferiority hypotheses. Institutional review board approval was not required for this study because only deidentified data were used. 

Both trials measured best-corrected visual acuity (BCVA) as letters read using the Early Treatment Diabetic Retinopathy Study (ETDRS) chart and harmonized definitions for measuring retinal thickness from OCTs, performed by designated reading centers (Duke OCT reading center in CATT; netWORC UK in the IVAN trial).^8,10^ Foveal center point thickness (FCPT) included the thickness of the neurosensory retina, subretinal fluid, and any subretinal hyperreflective material.^8,10^ In CATT, FCPT was measured at baseline and at 3, 6, 12, 18, and 24 months for participants in the monthly treatment group. In the PRN group of CATT, OCT grading of FCPT thickness was performed monthly. In the IVAN trial, FCPT was measured at baseline and every 3 months for all participants regardless of assignment to treatment regimen and at other visits if treatment failure criteria were met. Both trials used time-domain or spectral-domain OCTs. The proportions of each type of OCT scan used to measure FCPTs (from which FCPT SDs were calculated) are shown by trial in eTable 1 in the Supplement. In both trials, lesion size was measured using fluorescein angiography.

In this study, we consider the term *macular atrophy* to be synonymous with *geographic atrophy* (GA), which was graded and described in previous CATT and IVAN publications.^11^ Details of the methods used in each trial to grade fibrosis and GA are described in the eMethods in the Supplement.

The IVAN trial is registered^14^ and was approved by the National Research Ethics Committee, which covered all participating sites. The trial complied with the European Union Clinical Trials Directive 2001. CATT is registered^15^ and was approved by an institutional review board at each center and was performed in compliance with the Health Insurance Portability and Accountability Act. All participants provided written informed consent. Both trials adhered to the principles of the Declaration of Helsinki.

### Outcome Measures

The primary outcome for this analysis was BCVA in the study eye at the final 2-year visit or the exit visit for participants who withdrew before 2 years when all investigations scheduled for the 2-year visit were carried out. Secondary outcomes were the development of new fibrosis and GA during follow-up.

### Study Population

The study population included all participants from the IVAN and CATT trials. Participants with 3 or fewer FCPT measurements were excluded from the analysis population.

### Statistical Analyses

The objectives of these post hoc analyses were documented in advance of carrying out any analyses, although the inclusion of GA as an outcome was added at a later stage. We computed the SD of repeated FCPT measurements for each study eye across the entire duration of the trial. Study eyes were then categorized by FCPT SD quartile, ranging from low FCPT SD (quartile 1) to high FCPT SD (quartile 4). Participant demographic characteristics, mean study eye BCVA at baseline and final visit, baseline FCPT, and nAMD lesion characteristics are summarized by FCPT SD quartile.

We estimated the association of study eye FCPT SD quartile with BCVA at final visit using linear regression, adjusting for baseline BCVA, trial, and randomized allocations to drug and treatment regimen. We estimated the associations of FCPT SD quartile with the development of fibrosis and GA in eyes that did not exhibit these features at baseline using logistic regression, adjusting for randomized trial allocations.

Four sensitivity analyses were performed (eMethods in the Supplement):

1. Restricting the model to participants who had 9 or more FCPT measurements during time of study.
2. Adjusting the model additionally for age, lesion size, choroidal neovascularization type (classic vs occult), FCPT, and intraretinal fluid (IRF) at baseline.
3. Restricting the analyses to the groups allocated to treatment when required.
4. Censoring follow-up at 1 year if fibrosis developed during the first year.

Three additional analyses were performed to (1) explore whether the association of FCPT SD quartile with outcome differed between study eyes with a high FCPT compared with those with a low FCPT; (2) describe the association of injection frequency with variation in retinal thickness, restricted to the PRN groups (same rationale as for sensitivity analysis 3); and (3) contrast the associations by treatment regimen, fitting the interaction of FCPT SD quartile and trial treatment regimen allocation.

Associations are reported as effect estimates with 95% CIs; we made no adjustment for multiple estimation. We tested interactions of FCPT SD quartile and trial using likelihood ratio tests in each regression and report associations separately by trial when the interaction had a *P* value of .10 or less (2-tailed). In all tables, the numbers of missing data are described in footnotes. Further details of the analyses are described in the eMethods in the Supplement. All analyses were performed using Stata version 15.1 (StataCorp).

## Results

### Study Population

A total of 1185 participants recruited to CATT and 610 participants recruited to the IVAN trial were eligible for inclusion. We extracted the FCPT measurements for 1165 participants from CATT and 566 participants from the IVAN trial with 4 or more FCPT measurements. Of the 1731 included patients, 1058 (61.1%) were female, and the mean (SD) age was 78.6 (7.4) years.

FCPT SD is shown by trial and randomized allocations in eTable 2 in the Supplement. The median (interquartile range) FCPT SD was 40.2 (27.1-61.2) in the IVAN cohort and 59.0 (38.3-89.4) in the CATT cohort. Box plots of the FCPT SD distributions are shown in eFigure 1 in the Supplement. The FCPT SD values among study eyes were less than 34.01 μm in quartile 1, 34.01 μm to less than 51.49 μm in quartile 2, 51.49 μm to less than 80.59 μm in quartile 3, and greater than 80.59 μm in quartile 4. B scans at each follow-up visit for a representative study eye in quartile 1 and quartile 4 are shown in eFigure 2 in the Supplement. There was no statistically significant interaction for FCPT SD quartile with trial identity in any of the primary models. FCPT at quarterly intervals is summarized by FCPT SD quartile in eTable 3 in the Supplement. The distributions of treatment frequency by treatment regimen are shown in eFigure 3 in the Supplement.

Table 1 shows participant demographic characteristics and frequencies of markers of systemic health, and eTable 4 in the Supplement shows baseline morphology by FCPT SD quartile for the combined CATT and IVAN population. BCVA by FCPT SD quartile at baseline, final visit, and change from baseline is shown in Table 2 for the combined population and by trial. At both the baseline and final visits, BCVA was highest in quartile 1, decreasing steadily across quartiles. Similar findings were seen in each trial population. A scatterplot of FCPT SD vs BCVA at the final visit (eFigure 4 in the Supplement) shows decreasing BCVA with increasing FCPT SD.

**Table 1.** Participant Demographic Characteristics and History at Baseline by Foveal Center Point Thickness (FCPT) SD Quartile

Characteristic	No./total No. (%)				
	Quartile 1 (n = 433)	Quartile 2 (n = 433)	Quartile 3 (n = 433)	Quartile 4 (n = 432)	Overall (n = 1731)
**Demographic characteristics**					
Age, mean (SD), y	77.7 (7.5)	78.6 (7.2)	79.0 (7.3)	79.1 (7.4)	78.6 (7.4)
Male	162/433 (37.4)	175/433 (40.4)	171/433 (39.5)	165/432 (38.2)	673/1731 (38.9)
Blood pressure, mean (SD), mm Hg					
Systolic	136.9 (17.8)	137.9 (18.5)	137.9 (18.8)	136.9 (19.3)	137.4 (18.6)
Diastolic	76.3 (9.8)	76.0 (10.0)	76.4 (10.1)	74.6 (9.9)	75.8 (10.0)
Baseline lesion size, median (IQR), mm^2^^a^	3.3 (1.4-6.9)	3.7 (1.8-8.3)	4.7 (2.2-8.9)	6.5 (3.1-11.6)	4.4 (1.9-8.8)
**Nonocular history**					
Angina	42/433 (9.7)	54/433 (12.5)	41/433 (9.5)	51/432 (11.8)	188/1731 (10.9)
Dyspnea^b^	39/220 (17.7)	28/145 (19.3)	20/122 (16.4)	14/77 (18.2)	101/564 (17.9)
Asthma^c^	27/213 (12.7)	33/286 (11.5)	25/311 (8.0)	38/355 (10.7)	123/1165 (10.6)
Cough/wheeze^c^	36/213 (16.9)	62/286 (21.7)	45/311 (14.5)	73/355 (20.6)	216/1165 (18.5)
Emphysema^c^	8/213 (3.8)	19/286 (6.6)	19/311 (6.1)	24/355 (6.8)	70/1165 (6.0)
MI	36/433 (8.3)	52/433 (12.0)	44/433 (10.2)	45/432 (10.4)	177/1731 (10.2)
Transient ischemic attack	16/412 (3.9)	29/429 (6.8)	24/428 (5.6)	26/430 (6.0)	95/1699 (5.6)
Stroke	17/433 (3.9)	20/433 (4.6)	24/433 (5.5)	20/432 (4.6)	81/1731 (4.7)
DVT/PE^b^	23/220 (10.5)	5/147 (3.4)	4/121 (3.3)	13/77 (16.9)	45/565 (8.0)
Phlebitis/blood clots^c^	13/213 (6.1)	16/286 (5.6)	9/311 (2.9)	16/355 (4.5)	54/1165 (4.6)
Current or past smoker	245/431 (56.8)	255/430 (59.3)	261/433 (60.3)	261/431 (60.6)	1022/1725 (59.2)

Abbreviations: DVT, deep vein thrombosis; IQR, interquartile range; MI, myocardial infarction; PE, pulmonary embolism.

^a^ Data missing for 67 participants, including 25 in quartile 1, 10 in quartile 2, 16 in quartile 3, and 16 in quartile 4.

^b^ Data available from the Inhibition of VEGF in Age-Related Choroidal Neovascularization trial only.

^c^ Data available for the Comparison of Age-Related Macular Degeneration Treatments Trials only.

**Table 2.** Mean Best-Corrected Visual Acuity at Baseline and Final Visit by Foveal Center Point Thickness SD Quartile^a^

Trial	Mean (SD)				
	Quartile 1 (n = 433)	Quartile 2 (n = 433)	Quartile 3 (n = 433)	Quartile 4 (n = 432)	Overall (n = 1731)
**Overall**					
No.	433	433	433	432	1731
Baseline	66.8 (12.9)	61.9 (12.9)	60.8 (13.1)	54.6 (14.4)	61.0 (14.0)
Final visit^b^	72.8 (14.4)	67.6 (17.4)	66.8 (17.3)	59.0 (21.4)	66.5 (18.5)
Change from baseline^b^	6.0 (13.2)	5.6 (15.2)	5.9 (14.6)	4.4 (20.2)	5.5 (16.0)
**IVAN trial**					
No.	220	147	122	77	566
Baseline	66.2 (14.2)	60.4 (14.9)	60.7 (14.7)	53.8 (15.1)	61.8 (15.1)
Final visit^c^	71.2 (15.5)	63.5 (18.7)	65.9 (17.1)	56.7 (20.7)	66.1 (18.1)
Change from baseline^c^	5.0 (13.2)	3.1 (16.2)	5.2 (14.0)	2.9 (16.3)	4.2 (14.6)
**CATT**					
No.	213	286	311	355	1165
Baseline	67.5 (11.5)	62.7 (11.7)	60.8 (12.4)	54.7 (14.3)	60.7 (13.4)
Final visit^d^	74.5 (13.1)	69.6 (16.4)	67.1 (17.4)	59.5 (21.6)	66.7 (18.7)
Change from baseline^d^	7.0 (13.1)	6.9 (14.6)	6.2 (14.9)	4.7 (20.9)	6.1 (16.6)

Abbreviations: CATT, Comparison of Age-Related Macular Degeneration Treatments Trials; IVAN, Inhibition of VEGF in Age-Related Choroidal Neovascularization.

^a^ Visual acuity was measured using the Early Treatment Diabetic Retinopathy Study chart. Five letters is equivalent to 1 line on the Early Treatment Diabetic Retinopathy Study chart. Approximate Snellen equivalents for letter scores are: 75 letters, 20/30; 70 letters, 20/40; 65 letters, 20/50; 60 letters, 20/60; 55 letters, 20/80; and 50 letters, 20/100.

^b^ Data missing for 6 participants, including 2 from quartile 1, 2 from quartile 2, 1 from quartile 3, and 1 from quartile 4.

^c^ Data missing for 1 participant, including 1 from quartile 2.

^d^ Data missing for 5 participants, including 2 from quartile 1, 2 from quartile 3, and 1 from quartile 4.

**Figure 1.**
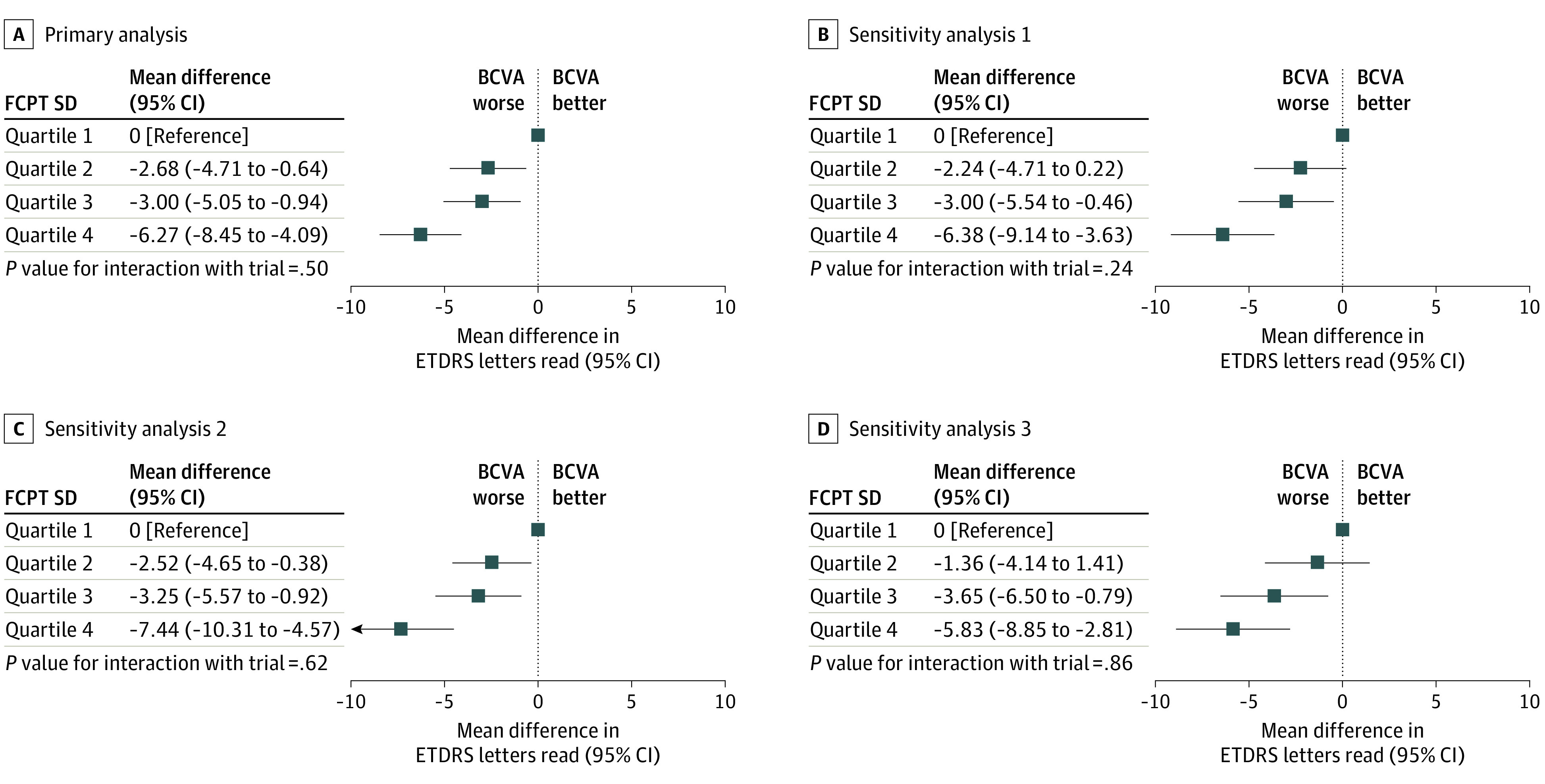
Estimates of Associations of Foveal Center Point Thickness (FCPT) SD Quartile With Final Best-Corrected Visual Acuity (BCVA) A, The primary analysis model adjusted for baseline BCVA, trial, and randomized allocations to drug and treatment regimen and included 1720 participants. B, Sensitivity analysis 1, which restricted the primary model to participants with 9 or more FCPT measurements, included 1169 participants. C, Sensitivity analysis 2, which additionally adjusted the primary model for age, baseline legion size, choroidal neovascularization type, FCPT, and intraretinal fluid, included 1577 participants with complete data. D, Sensitivity analysis 3, which restricted the primary model to participants in the pro re nata groups only, included 870 participants. Quartile 1 was defined as an FCPT SD less than 34.01 μm; quartile 2, 34.01 μm to less than 51.49 μm; quartile 3, 51.49 μm to less than 80.59 μm; and quartile 4, greater than 80.59 μm. ETDRS indicates Early Treatment Diabetic Retinopathy Study.

Using quartile 1 as the reference category, there was a strong association of FCPT SD quartile with the estimated difference in BCVA at the final visit **(**Figure 1A) (n = 1720; quartile 2, −2.68; 95% CI, −4.71 to −0.64; quartile 3, −3.00; 95% CI, −5.05 to −0.94; quartile 4, −6.27; 95% CI, −8.45 to −4.09), adjusted for baseline BCVA and randomized allocations. Sensitivity analysis 1 (Figure 1B), sensitivity analysis 2 (Figure 1C), sensitivity analysis 3 (Figure 1D), and sensitivity analysis 4 (eFigure 5 in the Supplement) showed very similar associations. The first additional analysis (primary model) confirmed that the association of FCPT SD quartile with BCVA was consistent across strata when study eyes were stratified by low vs high average FCPT (eFigure 6 in the Supplement). The interaction of FCPT SD and treatment regimen showed similar associations (direction and gradient) with BCVA for both monthly and PRN regimens (eFigure 7 in the Supplement).

Data on the presence of fibrosis were available at both the baseline and final visits in 1578 participants (1061 participants from CATT and 517 participants from the IVAN trial). The proportion of eyes with fibrosis rose from 7.8% (135 of 1720) at baseline to 58.7% (931 of 1586) at the final visit. The frequencies of eyes with fibrosis observed at baseline and eyes that developed fibrosis by final visit are summarized by quartile of FCPT SD in eTable 5 in the Supplement. By the final visit, 789 of 1443 study eyes (54.7%) that did not have fibrosis at baseline had developed fibrosis. The proportion of eyes developing fibrosis by the final visit was highest in quartile 4 and lowest in quartile 1 (eTable 5 in the Supplement).

**Figure 2.**
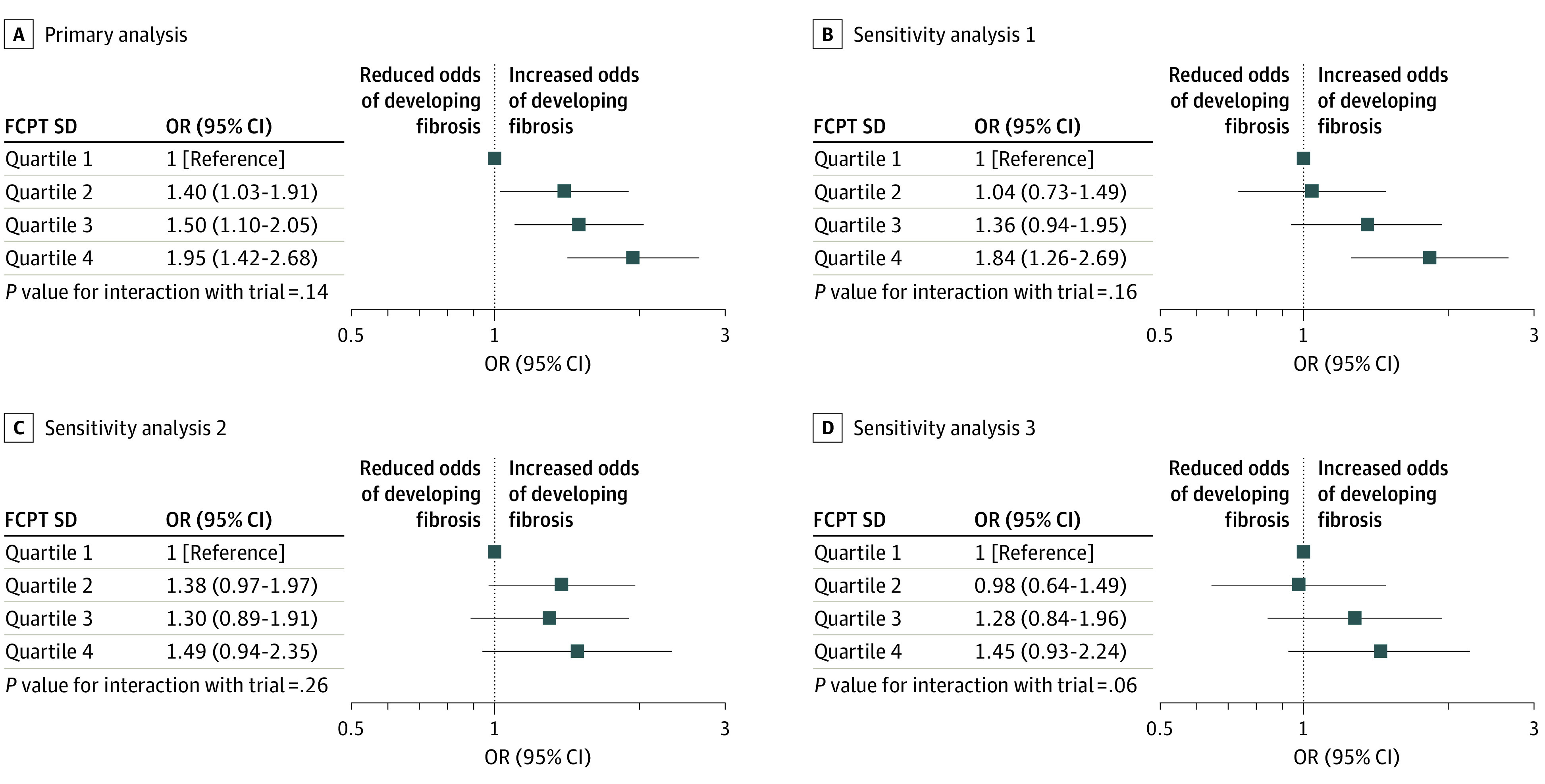
Estimates of Associations of Foveal Center Point Thickness (FCPT) SD Quartile With Development of Fibrosis Models were restricted to participants with fibrosis absent at baseline and data available at final visit (n = 1443). A, The primary analysis model adjusted for baseline trial and randomized allocations to drug and treatment regimen and included 1443 participants, of which 789 developed fibrosis. B, Sensitivity analysis 1, which restricted the primary model to participants with 9 or more FCPT measurements, included 1007 participants. C, Sensitivity analysis 2, which additionally adjusted the primary model for age, baseline legion size, choroidal neovascularization type, FCPT, and intraretinal fluid, included 1335 participants with complete data. D, Sensitivity analysis 3, which restricted the primary model to participants in the pro re nata groups only, included 718 participants. Effect estimates by trial for sensitivity analysis 3 are described in eFigure 9 in the Supplement, as the interaction with trial was statistically significant. Quartile 1 was defined as an FCPT SD less than 34.01 μm; quartile 2, 34.01 μm to less than 51.49 μm; quartile 3, 51.49 μm to less than 80.59 μm; and quartile 4, greater than 80.59 μm. OR indicates odds ratio.

The odds of developing fibrosis increased with increasing variation in FCPT SD; after adjustment for original trial allocations, odds ratios ranged from 1.40 (95% CI, 1.03 to 1.91) for quartile 2 to 1.95 (95% CI, 1.42 to 2.68) for quartile 4 (Figure 2A). The overall findings of the sensitivity analyses were consistent with the primary analysis (Figure 2B-D; eFigure 8 in the Supplement), although there appeared to be an interaction by trial in sensitivity analysis 3. Effect estimates for the 2 trials separately for sensitivity analysis 3 are shown in eFigure 9 in the Supplement. The association of increasing FCPT SD with the development of fibrosis also differed when study eyes were stratified by low vs high average FCPT (eFigure 10 in the Supplement), with the strongest association in the group with high average FCPT. The interaction of FCPT SD and treatment regimen showed similar associations (direction and gradient) with the development of fibrosis for both monthly and PRN regimens (eFigure 11 in the Supplement).

**Figure 3.**
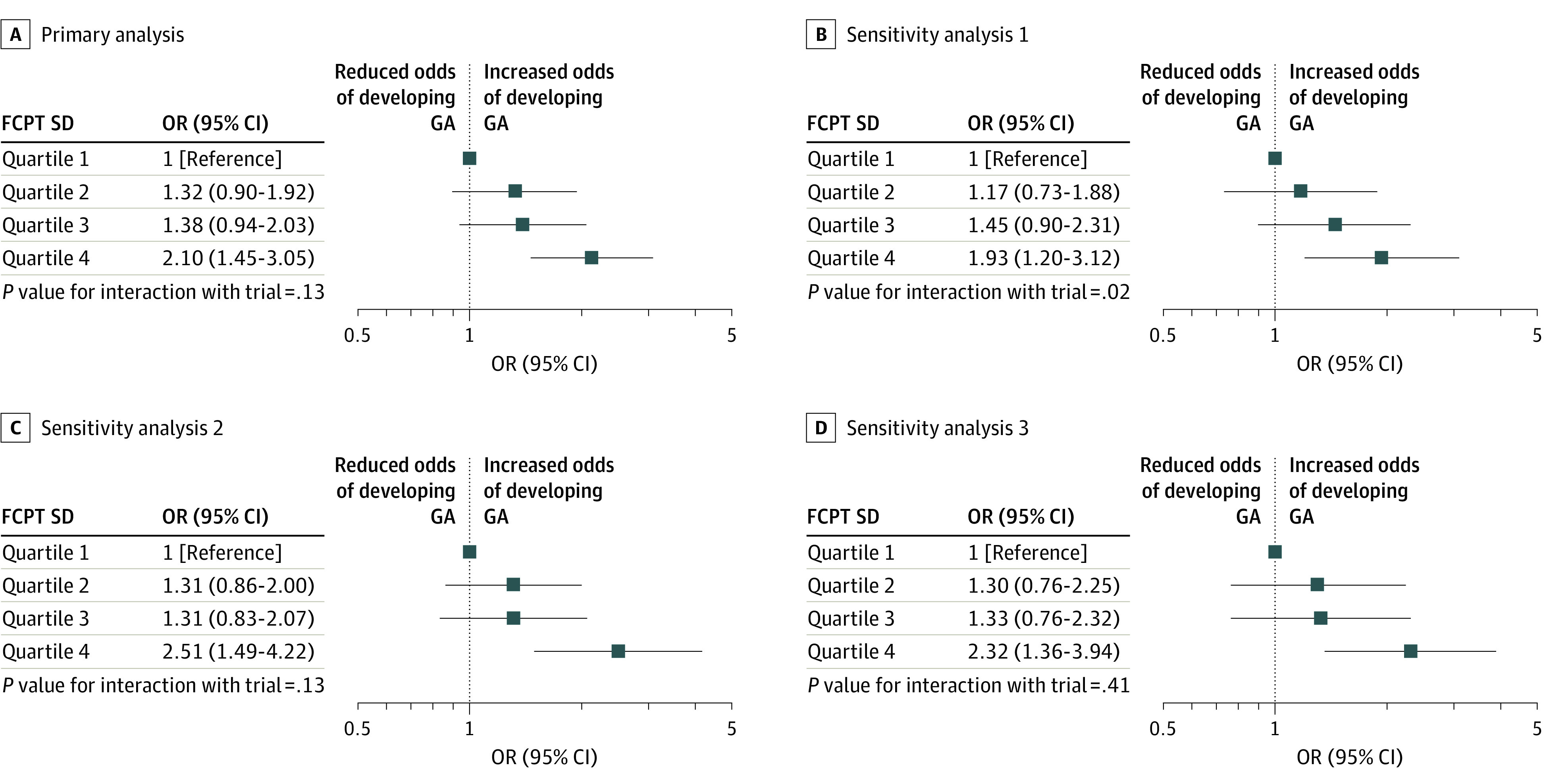
Estimates of Association of Foveal Center Point Thickness (FCPT) SD Quartile With Development of Geographic Atrophy (GA) Models were restricted to participants with GA absent at baseline and data available at final visit (n = 1463). A, The primary analysis model adjusted for baseline trial and randomized allocations to drug and treatment regimen and included 1463 participants, of which 310 developed GA. B, Sensitivity analysis 1, which restricted the primary model to participants with 9 or more FCPT measurements, included 1001 participants. C, Sensitivity analysis 2, which additionally adjusted the primary model for age, baseline legion size, choroidal neovascularization type, FCPT, and intraretinal fluid, included 1354 participants with complete data. D, Sensitivity analysis 3, which restricted the primary model to participants in the pro re nata groups only, included 734 participants. Quartile 1 was defined as an FCPT SD less than 34.01 μm; quartile 2, 34.01 μm to less than 51.49 μm; quartile 3, 51.49 μm to less than 80.59 μm; and quartile 4, greater than 80.59 μm. OR indicates odds ratio.

A total of 155 of 1726 study eyes (9.0%) had GA at baseline (quartile 1, 49 of 431 [11.4%]; quartile 2, 41 of 432 [9.5%]; quartile 3, 43 of 432 [10.0%]; quartile 4, 22 of 431 [5.1%]). By the final visit, 310 of 1463 study eyes (21.2%) that did not have GA at baseline had developed GA. The proportion of participants developing GA by the final visit was highest in participants in quartile 4 (103 of 383 [26.9%]) and lowest in quartile 1 (63 of 367 [17.2%])_._ The odds of developing GA increased across FCPT SD quartiles; after adjustment for randomized allocations, odds ratios ranged from 1.32 (95% CI, 0.90 to 1.92) for quartile 2 to 2.10 (95% CI, 1.45 to 3.05) for quartile 4 (Figure 3). Sensitivity analyses showed associations in the same direction and similar gradients across quartiles (Figure 3) (eFigures 12 and 13 in the Supplement).

The additional analysis of the association of number of injections with FCPT SD showed that, after adjustment for drug allocation, number of injections was positively associated with increasing odds of an eye being classified in a higher FCPT SD quartile (eTable 6 in the Supplement). For every 3 additional injections, the odds of being in a higher FCPT SD quartile was 13% (95% CI, 7 to 20) higher. This association was unaltered by adjusting for baseline lesion size.

## Discussion

In this study, after 2 years of anti-VEGF therapy, eyes with greater fluctuation in retinal thickness had worse BCVA and were more likely to develop fibrosis and GA in the macular lesion than eyes that had less fluctuation. We chose to use data from the CATT and IVAN trials as these trials were undertaken contemporaneously comparing the same 2 anti-VEGF agents^8,9,10,11^ and monthly vs PRN treatment regimens. Both trials withheld treatment in PRN groups when retreatment criteria were not met. In CATT, eyes with fluid were to be treated unless the ophthalmologist chose to stop treatment for futility. This determination could be made after 3 consecutive monthly injections with no decrease in fluid. Futility was invoked in less than 3% of participants. In the IVAN trial, shallow pigment epithelial elevation was tolerated if there was no subretinal or IRF or if the pigment epithelial detachment had not increased since the prior visit. Both trials reviewed participants monthly, with good retention and only about 5% of visits being missed. Hence, we were able to model the association of retinal thickness fluctuation without the confounding effects of suboptimal treatment that can occur in clinical practice, eg, clinic cancellations or patient-related issues.

Our primary analyses only adjusted for BCVA at baseline and randomized allocations and found a difference of more than 6 ETDRS letters (about 1 Snellen line) in BCVA at the final visit between quartile 1 and quartile 4 of FCPT SD, the difference increasing smoothly across quartiles. In sensitivity analyses, we adjusted for age, lesion size, classic choroidal neovascularization, IRF, and baseline FCPT and excluded the monthly treatments groups. All sensitivity analyses showed the same pattern of results as the primary analyses. Assuming that quartile 1 reflects a persistently fluid-free state or very low levels of retinal thickness fluctuation and quartile 4 reflects episodic retinal thickening due to reaccumulation of fluid at some visits, to our knowledge, our analyses demonstrate for the first time clear differences in BCVA outcome between these states, in the optimal follow-up and treatment setting of randomized trials.

Tolerating small amounts of IRF, subretinal fluid, and subretinal pigment epithelium fluid in the macula has been a topic of controversy for some time; maintaining the macula free of fluid is the basis of the treat-and-extend approach.^16,17,18^ Treat and extend requires administration of treatment even when the macula is free of fluid at review, assuming that recurrence of lesion activity with even low degrees of leakage may cause unrecoverable vision loss. Arguments against this approach include unnecessary risk to the patient from endophthalmitis,^1^ longer-term risks such as macular atrophy,^19^ and the recognition that the presence of shallow subretinal fluid, which may contain beneficial growth factors, is associated with a better outcome.^20,21^

Two important factors determining visual outcome in eyes receiving anti-VEGF therapy are the onset of fibrosis and GA, and worse outcomes have been reported when nAMD lesions exhibit these features.^22,23,24,25,26^ A shift in the balance between VEGF and connective tissue growth factor has been identified as a predisposing factor in the development of fibrosis.^27^ However, even before the introduction of anti-VEGF therapies, fibrosis was reported at a high frequency in patients with chronic nAMD lesions.^28^ The higher FCPT SD could be viewed as a proxy measure for bouts of worsening that occur in conjunction with the cyclical treatment paradigms that were established when anti-VEGF agents came into clinical use.^29^ It is notable that in nonocular tissues, intermittent stretch is known to result in the recruitment of macrophages that trigger fibrosis.^30^ Several other biological mechanisms promote fibrosis, and it is possible that the angiofibrotic switch is more strongly activated in eyes with greater retinal thickness fluctuation.

Several risk factors have been reported to be associated with a higher incidence of GA in the context of treated nAMD.^21,31,32^ Incident GA was also more likely to occur in eyes with the highest FCPT SD. Eyes with worse nAMD disease at baseline, reflected by a larger lesion size and greater retinal thickness, may have lost more neural tissue and thus been more prone to developing features of atrophy.

### Strengths and Limitations

Our study has several strengths. Data and images were collected according to trial protocols and were largely complete, and images were graded independently with masking. Data were from 2 trials, both of which were multicenter studies, enhancing the applicability of our findings. Analyses were consistent across several sensitivity analyses.

Our study also has limitations. The analyses were post hoc in that they were not planned in advance of starting the trials, although the objectives were prespecified. We required a measure that reflected macular thickness changes over the entire follow-up in any given participant. Macular volume would have been the ideal measurement, but this was not available in either trial. Our proxy outcome was FCPT, recorded accurately at least every 3 months; the intraclass correlation coefficient for repeated grading of FCPT in CATT was 0.99, with 95% limits of agreement on the difference between gradings of −55 to 47 um. Both trials were conducted between 2007 and 2012, and therefore, some participants underwent imaging using spectral-domain OCT and some with time-domain OCT instruments, the latter with poorer quality resolution compared with the former. We addressed the differences in image acquisition through application of conversion factors by the grading centers.

Within the PRN arm, contributions of variation in retinal thickness and treatment frequency cannot be separated. However, associations of FCPT SD with outcomes were consistent for continuous and PRN regimens, supporting the view that retinal thickness was the important driver. Other limitations include variable amounts of missing data across participants and the potential for residual confounding. The former was addressed by sensitivity analysis 1 and the latter by sensitivity analysis 2.

## Conclusions

The finding that increasing variation in retinal thickness was adversely associated with BCVA and the risk of developing fibrosis and GA provides an impetus to seek agents with greater treatment durability or sustained release devices, such as those currently undergoing evaluation.^5^ In summary, the findings of the present analyses are clinically important with respect to prognosis in nAMD and offer insights into key functional and morphological outcomes in patients with nAMD undergoing treatment anti-VEGF agents.

## Notes

Supplement. **eMethods.** **eTable 1.** Type of ocular coherence tomography scan used for FCPT measurement. **eTable 2.** Within participant FCPT SD by trial and by randomized allocations. **eTable 3.** Median FCPT during trial by FCPT SD quartile. **eTable 4.** Baseline morphology by FCPT SD quartile. **eTable 5.** Presence of fibrosis in study eye at baseline and follow-up by FCPT SD quartile. **eTable 6.** Post hoc analysis: association of total number of injections with FCPT SD (discontinuous/PRN group only). **eFigure 1.** Boxplot of within-participant FCPT SD by randomized allocations. **eFigure 2.** Trial OCT scans of representative participants in FCPT SD quartile 1 and FCPT SD quartile 4. **eFigure 3.** Boxplot of the number of injections received by randomized allocations. **eFigure 4.** BCVA at final visit by FCPT SD. **eFigure 5.** Sensitivity analysis 4: estimates of associations of FCPT SD quartile with final BCVA. **eFigure 6.** Estimates of associations of FCPT SD quartile with final BCVA by low/high average FCPT (interaction). **eFigure 7.** Estimates of associations of FCPT SD quartile with final BCVA by treatment regimen trial allocation (interaction). **eFigure 8.** Sensitivity analysis 4: estimates of associations of FCPT SD quartile with development of fibrosis. **eFigure 9.** Sensitivity analysis 3 by trial: estimates of associations of FCPT SD quartile with development of fibrosis. **eFigure 10.** Estimates of associations of FCPT SD quartile with development of fibrosis by low/high average FCPT (interaction). **eFigure 11.** Estimates of associations of FCPT SD quartile with development of fibrosis by treatment regimen trial allocation (interaction). **eFigure 12.** Sensitivity analysis 4: estimates of associations of FCPT SD quartile with development of geographic atrophy. **eFigure 13.** Estimates of associations of FCPT SD quartile with development of geographic atrophy by treatment frequency allocation (interaction).
