# Supplementary material for: Associations of Variation in Retinal Thickness With Visual Acuity and Anatomic Outcomes in Eyes With Neovascular Age-Related Macular Degeneration Lesions Treated With Anti–Vascular Endothelial Growth Factor Agents
Source: JAMA Ophthalmol. 2020 Aug 20;138(10):1043–51. doi: 10.1001/jamaophthalmol.2020.3001 (PMC7441468; doi:10.1001/jamaophthalmol.2020.3001)
Supplement: Supplement. — eMethods. eTable 1. Type of ocular coherence tomography scan used for FCPT measurement. eTable 2. Within participant FCPT SD by trial and by randomized allocations. eTable 3. Median FCPT during trial by FCPT SD quartile. eTable 4. Baseline morphology by FCPT SD quartile. eTable 5. Presence of fibrosis in study eye at baseline and follow-up by FCPT SD quartile. eTable 6. Post hoc analysis: association of total number of injections with FCPT SD (discontinuous/PRN group only). eFigure 1. Boxplot of within-participant FCPT SD by randomized allocations. eFigure 2. Trial OCT scans of representative participants in FCPT SD quartile 1 and FCPT SD quartile 4. eFigure 3. Boxplot of the number of injections received by randomized allocations. eFigure 4. BCVA at final visit by FCPT SD. eFigure 5. Sensitivity analysis 4: estimates of associations of FCPT SD quartile with final BCVA. eFigure 6. Estimates of associations of FCPT SD quartile with final BCVA by low/high average FCPT (interaction). eFigure 7. Estimates of associations of FCPT SD quartile with final BCVA by treatment regimen trial allocation (interaction). eFigure 8. Sensitivity analysis 4: estimates of associations of FCPT SD quartile with development of fibrosis. eFigure 9. Sensitivity analysis 3 by trial: estimates of associations of FCPT SD quartile with development of fibrosis. eFigure 10. Estimates of associations of FCPT SD quartile with development of fibrosis by low/high average FCPT (interaction). eFigure 11. Estimates of associations of FCPT SD quartile with development of fibrosis by treatment regimen trial allocation (interaction). eFigure 12. Sensitivity analysis 4: estimates of associations of FCPT SD quartile with development of geographic atrophy. eFigure 13. Estimates of associations of FCPT SD quartile with development of geographic atrophy by treatment frequency allocation (interaction). [file jamaophthalmol-e203001-s001.pdf]

Supplementary Online Content

Evans RN, Reeves BC, Maguire MG, et al. Associations of variation in retinal thickness with visual acuity and anatomic outcomes in eyes with neovascular age-related macular degeneration lesions treated with anti–vascular endothelial growth factor agents. *JAMA Ophthalmol*. Published online August 20, 2020. doi:10.1001/jamaophthalmol.2020.3001

eMethods.

eTable 1. Type of ocular coherence tomography scan used for FCPT measurement

eTable 2. Within participant FCPT SD by trial and by randomized allocations

eTable 3. Median FCPT during trial by FCPT SD quartile

eTable 4. Baseline morphology by FCPT SD quartile

eTable 5. Presence of fibrosis in study eye at baseline and follow-up by FCPT SD quartile

eTable 6. Post hoc analysis: association of total number of injections with FCPT SD (discontinuous/PRN group only)

eFigure 1. Boxplot of within-participant FCPT SD by randomized allocations

eFigure 2. Trial OCT scans of representative participants in FCPT SD quartile 1 and FCPT SD quartile 4

eFigure 3. Boxplot of the number of injections received by randomized allocations

eFigure 4. BCVA at final visit by FCPT SD

eFigure 5. Sensitivity analysis 4: estimates of associations of FCPT SD quartile with final BCVA

eFigure 6. Estimates of associations of FCPT SD quartile with final BCVA by low/high average FCPT (interaction)

eFigure 7. Estimates of associations of FCPT SD quartile with final BCVA by treatment regimen trial allocation (interaction)

eFigure 8. Sensitivity analysis 4: estimates of associations of FCPT SD quartile with development of fibrosis

eFigure 9. Sensitivity analysis 3 by trial: estimates of associations of FCPT SD quartile with development of fibrosis

eFigure 10. Estimates of associations of FCPT SD quartile with development of fibrosis by low/high average FCPT (interaction)

eFigure 11. Estimates of associations of FCPT SD quartile with development of fibrosis by treatment regimen trial allocation (interaction)

eFigure 12. Sensitivity analysis 4: estimates of associations of FCPT SD quartile with development of geographic atrophy

© 2020 Evans RN et al. *JAMA Ophthalmology.*

eFigure 13. Estimates of associations of FCPT SD quartile with development of geographic atrophy by treatment frequency allocation (interaction)

This supplementary material has been provided by the authors to give readers additional information about their work.

© 2020 Evans RN et al. *JAMA Ophthalmology.*

eMethods.

Grading methods - additional information

In IVAN, grading for fibrosis and GA, used color images and fluorescein angiograms (FA) at baseline, 12 and 24 months. Fibrosis was graded as present when creamy white or yellow material was observed within the boundaries of the nAMD lesion on color images. On FA, fibrosis was deemed to be present if there was an area of blocked fluorescence in the early phase with hyperfluorescence in the mid-phase which faded in late frames. GA was defined as any area of atrophy, present either in regions previously occupied by the nAMD complex or elsewhere. In color images GA is seen as a region of pallor with or without sharp edges leading to visibility of underlying choroidal large vessels. On FA, this corresponds to an area of early hyperfluorescence with well-defined borders which does not change during the angiographic run.

In CATT, fibrotic scars were defined as obvious white or yellow mounds of fibrous-appearing tissue that were well defined in shape and appeared solid on color stereo images. Hyperfluorescence due to tissue staining or blocked fluorescence of the underlying choroid was present on FA. Both CFP and FA were used in assessing and characterizing GA. The diagnosis of GA required the presence within the macular vascular arcades of ≥1 patches ≥250 μ in longest linear dimension of partial or complete depigmentation in the CFP that had ≥1 of these additional characteristics: sharply demarcated borders seen in CFP and/or FA, visibility of underlying choroidal vessels, excavated or punched out appearance on stereoscopy of CFP or FA, or uniform hyperfluorescence bounded by sharp borders on late-phase angiography.

Statistical methods - additional information

All analyses were restricted to eyes for which data for all model covariates were available and included trial as a fixed effect. An interaction term between $FCPT_{SD}$ quartile and trial was included to investigate whether the association differed between the studies. If the interaction was not statistically significant at the 10% level, we report an overall effect. If the interaction was statistically significant, we report the effect sizes for the two studies separately. Assumptions underpinning the statistical models were checked using standard methods (e.g. residual plots). If the assumptions were not satisfied, transformations or alternative methods were explored.

Three sensitivity analyses (SA) were carried out to test the robustness of the estimates from the model.

- SA1. Restricting the model to participants who had ≥9 FCPT measurements during time on study, to minimize the variation in precision of $FCPT_{SD}$ across participants in the analysis.
- SA2. Adjusting the model additionally for age, lesion size, choroidal neovascularization (CNV) type (classic/occult), FCPT and intraretinal fluid (IRF) at baseline, because these covariates are prognostic for BCVA and might confound the association.
- SA3. Restricting the analyses to the groups allocated to treatment-when-required, because in the monthly groups (a) the distribution of treatment frequency was highly skewed and (b) non-adherence to monthly treatment was likely to have arisen due to missed visits.
- SA4. Censoring FCPT measurements at one year in the calculation of $FCPT_{SD}$ in eyes which develop fibrosis during the first year, to limit the inclusion of FCPT fluctuations that occurred after the development of fibrosis.

To explore whether the association between $FCPT_{SD}$ quartile and outcome differed between study eyes with a high FPCT compared to those with a low FCPT, for each individual average FCPT measurements during follow-up was calculated (within-person mean). Study eyes were dichotomized to low or high average FCPT and an interaction between average FCPT group and $FCPT_{SD}$ quartile was included in the model.

© 2020 Evans RN et al. *JAMA Ophthalmology.*

eTable 1. Type of ocular coherence tomography scan used for FCPT measurement

	Trial		
**OCT type**	**IVAN (n=4,792)**	**CATT (n=20,757)**	**Overall (n=25,549)**
	**n (%)**	**n (%)**	**n (%)**
SD	1,364 (28.5%)	2,485 (12.0%)	3,849 (15.1%)
TD	3,428 (71.5%)	18,272 (88.0%)	21,700 (84.9%)

Abbreviations: TD=Time domain, SD= Spectral domain, OCT= Ocular coherence tomography scan, FCPT=Foveal center point thickness

© 2020 Evans RN et al. *JAMA Ophthalmology.*

eTable 2. Within participant FCPT SD by trial and by randomized allocations

Trial allocation		IVAN		CATT	
		n	Median (IQR)	n	Median (IQR)
Drug	Ranibizumab	290	43.4 (28.1, 65.3)	593	59.7 (39.5, 90.8)
	Bevacizumab	276	38.6 (26.8, 59.2)	572	57.6 (36.9, 89.2)
Treatment regimen	Continuous	280	39.7 (26.9, 70.0)	574	62.7 (39.1, 97.1)
	Discontinuous/PRN	286	40.7 (28.1, 63.8)	591	54.0 (36.9, 84.7)
**Total**		**566**	**40.2 (27.1, 61.2)**	**1165**	**59.0 (38.3, 89.4)**

Notes: Medians (IQR) are reported because the distribution of $FCPT_{SD}$ is skewed. Please note that the IQR are very similar across the 4 groups despite minor variations in the medians.
CATT participants in the continuous group who were re-randomized at 1 year are analyzed as per their original trial allocation (continuous).

Abbreviations: $FCPT_{SD}$=Within participant standard deviation of foveal center point thickness, IQR=Interquartile range, PRN=Pro re nata

© 2020 Evans RN et al. *JAMA Ophthalmology.*

eFigure 1. Boxplot of within-participant FCPT SD by randomized allocations

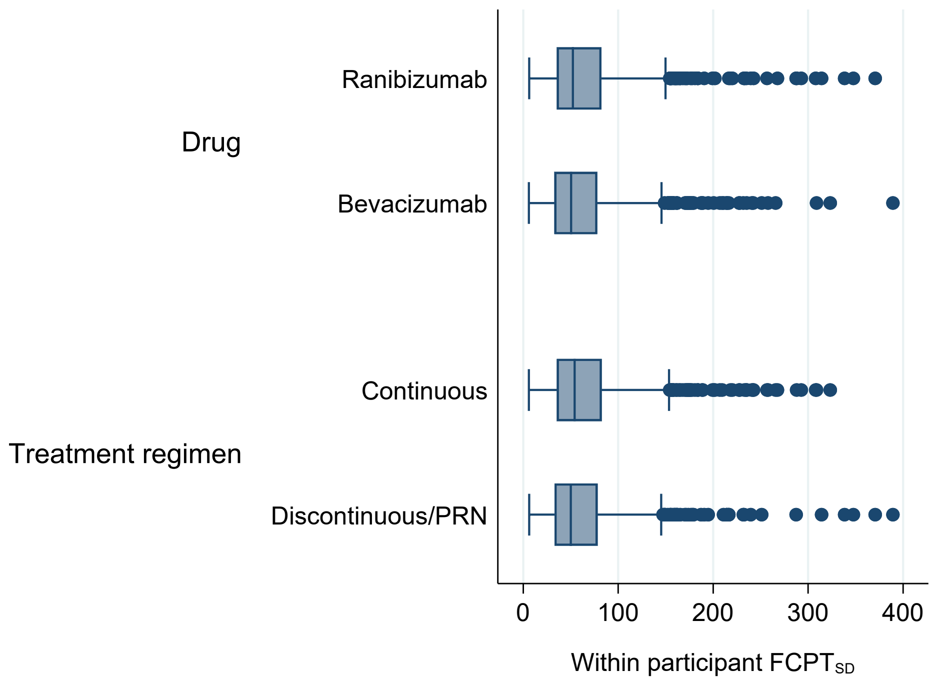

Notes: CATT participants in the continuous group who were re-randomized at 1 year are analyzed as per their original trial allocation (continuous).

Abbreviations: FCPT$_{SD}$= Within participant standard deviation of foveal center point thickness, PRN=Pro re nata

© 2020 Evans RN et al. *JAMA Ophthalmology.*

eFigure 2. Trial OCT scans of representative participants in FCPT SD quartile 1 and FCPT SD quartile 4

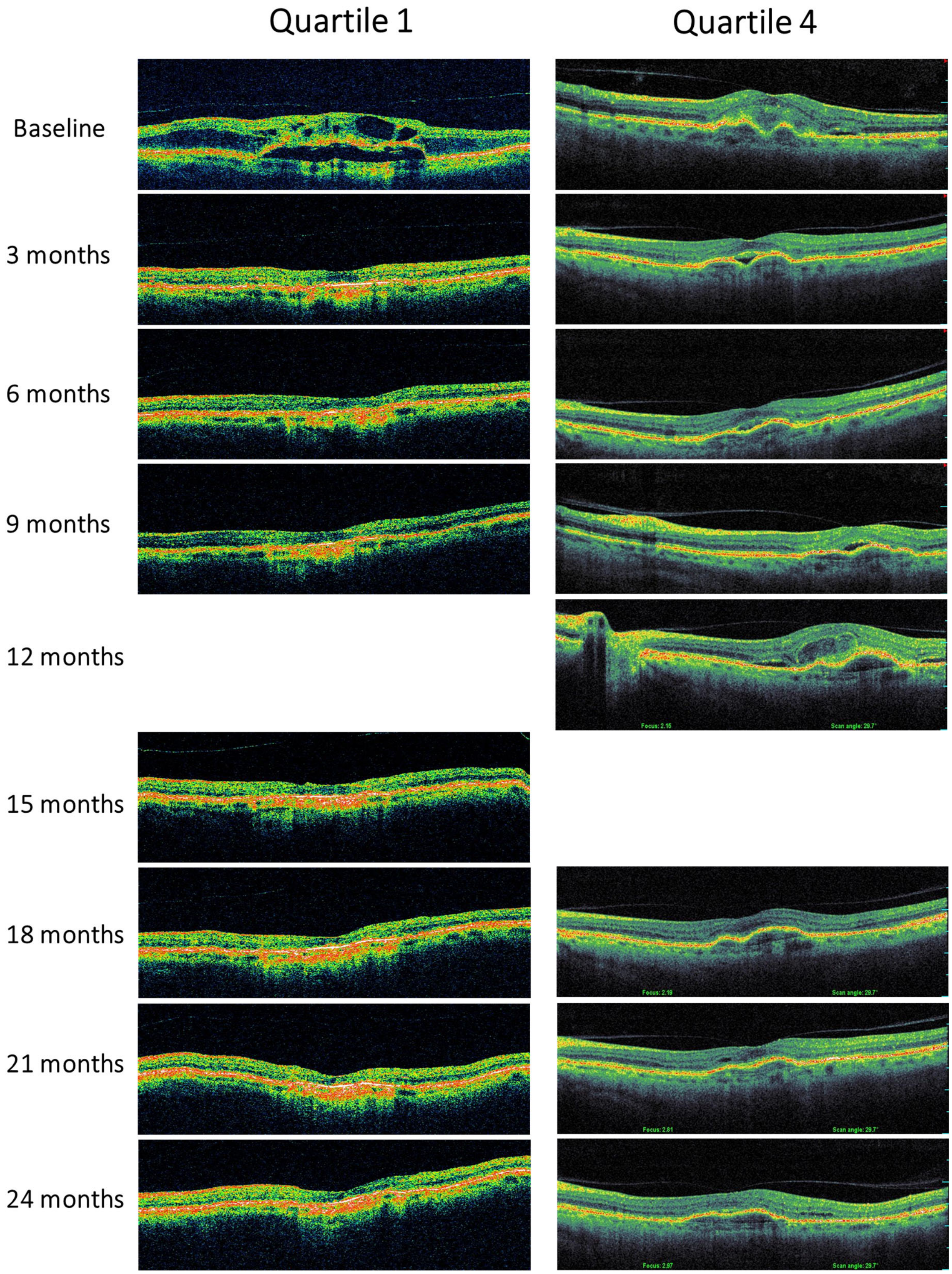

© 2020 Evans RN et al. *JAMA Ophthalmology.*

eTable 3. Median FCPT during trial by FCPT SD quartile

Trial		N	$FCPT_{SD}$ quartile Quartile 1 (n=433) (220 IVAN, 213 CATT)	Quartile 2 (n=433) (147 IVAN, 286 CATT)	Quartile 3 (n=433) (122 IVAN, 311 CATT)	Quartile 4 (n=432) (77 IVAN, 355 CATT)	Overall (n=1731) (566 IVAN, 1165 CATT)
		N	Median (IQR)	Median (IQR)	Median (IQR)	Median (IQR)	Median (IQR)
**IVAN (µm)**	Baseline[a]	566	320.0 (300.0, 350.0)	380.0 (360.0, 410.0)	445.0 (410.0, 480.0)	540.0 (470.0, 610.0)	380.0 (330.0, 440.0)
	3m[b]	562	300.0 (270.0, 325.0)	300.0 (270.0, 355.0)	300.0 (270.0, 380.0)	300.0 (270.0, 330.0)	300.0 (270.0, 340.0)
	6m[c]	565	300.0 (270.0, 330.0)	300.0 (270.0, 350.0)	300.0 (270.0, 360.0)	310.0 (280.0, 370.0)	300.0 (270.0, 340.0)
	9m[d]	563	300.0 (270.0, 320.0)	300.0 (270.0, 340.0)	300.0 (270.0, 340.0)	300.0 (280.0, 340.0)	300.0 (270.0, 330.0)
	12m[e]	551	300.0 (270.0, 330.0)	300.0 (270.0, 350.0)	300.0 (270.0, 360.0)	310.0 (270.0, 360.0)	300.0 (270.0, 340.0)
	15m[f]	542	300.0 (270.0, 330.0)	290.0 (270.0, 340.0)	300.0 (270.0, 330.0)	300.0 (270.0, 340.0)	300.0 (270.0, 330.0)
	18m[g]	529	300.0 (270.0, 330.0)	300.0 (270.0, 335.0)	290.0 (260.0, 330.0)	300.0 (270.0, 330.0)	300.0 (270.0, 330.0)
	21m[h]	522	300.0 (270.0, 320.0)	300.0 (270.0, 330.0)	300.0 (260.0, 340.0)	300.0 (260.0, 320.0)	300.0 (270.0, 330.0)
	24m[i]	518	290.0 (270.0, 320.0)	300.0 (270.0, 335.0)	300.0 (280.0, 345.0)	300.0 (280.0, 340.0)	300.0 (270.0, 330.0)
**CATT (µm)**	Baseline[j]	1165	280.5 (245.7, 325.4)	360.3 (313.5, 417.1)	441.8 (372.2, 531.7)	594.0 (478.5, 733.3)	418.0 (324.5, 555.5)
	3m[k]	1081	227.3 (198.0, 265.8)	256.7 (209.0, 328.2)	257.6 (209.0, 343.8)	297.9 (210.8, 476.7)	253.0 (207.2, 346.5)
	6m[l]	1050	227.3 (199.8, 264.0)	254.8 (211.8, 329.1)	265.8 (212.7, 366.7)	298.8 (207.2, 465.7)	256.7 (207.2, 355.7)
	9m[m]	504	236.5 (209.0, 275.0)	264.0 (214.5, 324.5)	281.4 (225.5, 357.5)	313.5 (221.8, 458.3)	269.5 (216.3, 353.8)
	12m[n]	1053	232.8 (198.0, 273.2)	249.3 (207.2, 320.8)	263.1 (205.3, 352.0)	267.7 (201.7, 418.0)	249.3 (203.5, 337.3)
	15m[o]	733	237.0 (209.0, 275.0)	264.0 (220.0, 341.0)	265.0 (214.5, 374.0)	302.5 (214.5, 462.0)	260.0 (214.5, 352.0)
	18m[p]	969	236.5 (203.5, 271.3)	271.3 (209.0, 324.5)	264.0 (214.5, 363.0)	282.3 (206.0, 424.0)	258.5 (209.0, 344.7)
	21m[q]	712	242.0 (214.5, 275.0)	264.0 (220.0, 335.5)	266.8 (212.5, 373.5)	299.8 (225.5, 434.5)	264.0 (216.0, 352.0)
	24m[r]	1023	236.5 (207.1, 272.2)	260.3 (220.0, 335.5)	258.5 (210.0, 374.0)	272.7 (197.1, 407.0)	253.9 (207.2, 349.0)

Notes: Medians (IQR) are reported because the distribution of FCPT is skewed.

© 2020 Evans RN et al. *JAMA Ophthalmology.*

[a] Data missing for 40 participants (22 Quartile 1, 6 Quartile 2, 10 Quartile 3, 2 Quartile 4)
[b] Data missing for 16 participants (7 Quartile 1, 3 Quartile 2, 5 Quartile 3, 1 Quartile 4)
[c] Data missing for 23 participants (10 Quartile 1, 6 Quartile 2, 3 Quartile 3, 4 Quartile 4)
[d] Data missing for 12 participants (3 Quartile 1, 4 Quartile 2, 3 Quartile 3, 2 Quartile 4)
[e] Data missing for 23 participants (6 Quartile 1, 5 Quartile 2, 9 Quartile 3, 3 Quartile 4)
[f] Data missing for 31 participants (12 Quartile 1, 6 Quartile 2, 3 Quartile 3, 10 Quartile 4)
[g] Data missing for 27 participants (6 Quartile 1, 6 Quartile 2, 7 Quartile 3, 8 Quartile 4)
[h] Data missing for 23 participants (8 Quartile 1, 6 Quartile 2, 5 Quartile 3, 4 Quartile 4)
[i] Data missing for 19 participants (6 Quartile 1, 4 Quartile 2, 6 Quartile 3, 3 Quartile 4)
[j] Data missing for 5 participants (1 Quartile 1, 2 Quartile 2, 1 Quartile 3, 1 Quartile 4)
[k] Data missing for 3 participants (0 Quartile 1, 0 Quartile 2, 2 Quartile 3, 1 Quartile 4)
[l] Data missing for 1 participant (0 Quartile 1, 0 Quartile 2, 0 Quartile 3, 1 Quartile 4)
[m] Data missing for 3 participants (0 Quartile 1, 1 Quartile 2, 1 Quartile 3, 1 Quartile 4)
[n] Data missing for 4 participants (0 Quartile 1, 1 Quartile 2, 2 Quartile 3, 1 Quartile 4)
[o] Data missing for 8 participants (0 Quartile 1, 1 Quartile 2, 2 Quartile 3, 5 Quartile 4)
[p] Data missing for 6 participants (0 Quartile 1, 1 Quartile 2, 2 Quartile 3, 3 Quartile 4)
[q] Data missing for 3 participants (1 Quartile 1, 0 Quartile 2, 0 Quartile 3, 2 Quartile 4)
[r] Data missing for 7 participants (0 Quartile 1, 2 Quartile 2, 1 Quartile 3, 4 Quartile 4)

Abbreviations: FCPT=Foveal center point thickness, $FCPT_{SD}$= Within participant standard deviation of foveal center point thickness, IQR=Interquartile range

© 2020 Evans RN et al. *JAMA Ophthalmology.*

eFigure 3. Boxplot of the number of injections received by randomized allocations

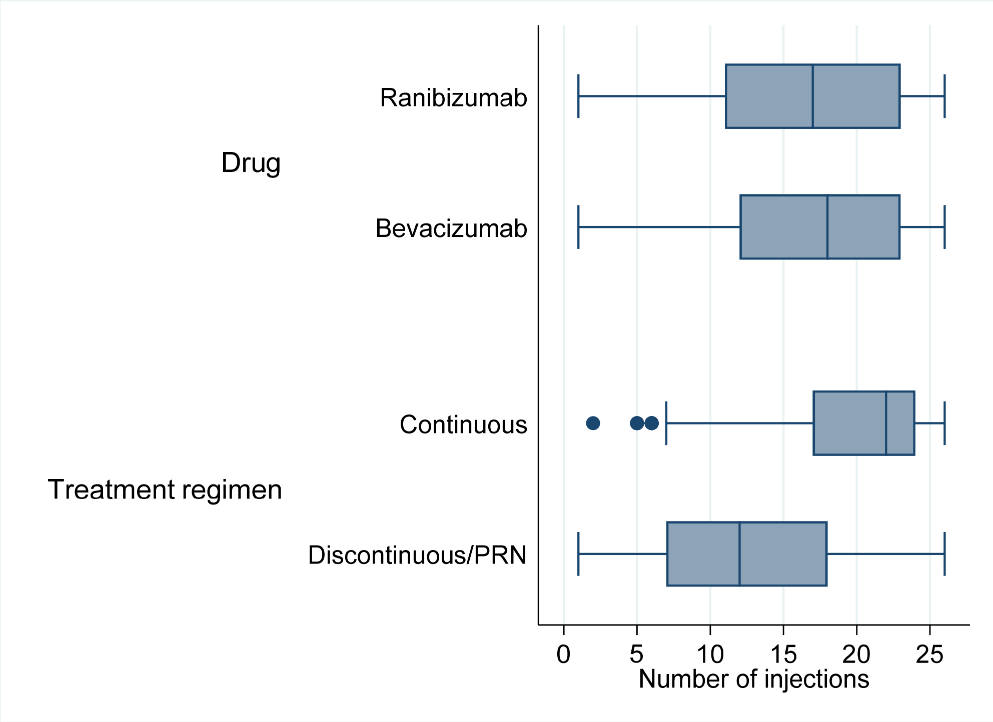

Notes: CATT participants in the continuous group who were re-randomized at 1 year are analyzed as per their original trial allocation (continuous).

Abbreviations: PRN=Pro re nata

© 2020 Evans RN et al. *JAMA Ophthalmology.*

eTable 4. Baseline morphology by FCPT SD quartile

Morphology feature	FCPT$_{SD}$ quartile				
	**Quartile 1 (n=433)**	**Quartile 2 (n=433)**	**Quartile 3 (n=433)**	**Quartile 4 (n=432)**	**Overall (n=1731)**
FCPT thickness, µm (mean, SD) [a]	308.0 (60.8)	377.1 (75.7)	458.2 (109.4)	611.0 (197.6)	440.1 (167.4)
Total lesion size, mm^2 (median, IQR) [b]	3.3 (1.4, 6.9)	3.7 (1.8, 8.3)	4.7 (2.2, 8.9)	6.5 (3.1, 11.6)	4.4 (1.9, 8.8)
Classic CNV (n, %)	96/409 (23.5%)	99/426 (23.2%)	106/423 (25.1%)	95/425 (22.4%)	396/1683 (23.5%)
GA (n, %)	49/431 (11.4%)	41/432 (9.5%)	43/432 (10.0%)	22/431 (5.1%)	155/1726 (9.0%)
Intra retinal fluid (n, %)	206/402 (51.2%)	275/421 (65.3%)	308/415 (74.2%)	365/425 (85.9%)	1154/1663 (69.4%)
SRF present (n, %)	316/406 (77.8%)	359/424 (84.7%)	355/420 (84.5%)	353/425 (83.1%)	1383/1675 (82.6%)
PED present (n, %) *	151/198 (76.3%)	106/140 (75.7%)	91/112 (81.3%)	63/74 (85.1%)	411/524 (78.4%)

* IVAN only
[a] *Data missing for 45 participants (23 Quartile 1, 8 Quartile 2, 11 Quartile 3, 3 Quartile 4)*
[b] *Data missing for 67 participants (25 Quartile 1, 10 Quartile 2, 16 Quartile 3, 16 Quartile 4)*

Abbreviations: FCPT=Foveal center point thickness, FCPT$_{SD}$= Within participant standard deviation of foveal center point thickness, SD=Standard deviation, IQR= Interquartile range, CNV= Choroidal neovascularization, GA=Geographic atrophy, SRF= Subretinal fluid, PED= Pigment Epithelial Detachment

© 2020 Evans RN et al. *JAMA Ophthalmology.*

eFigure 4. BCVA at final visit by FCPT SD

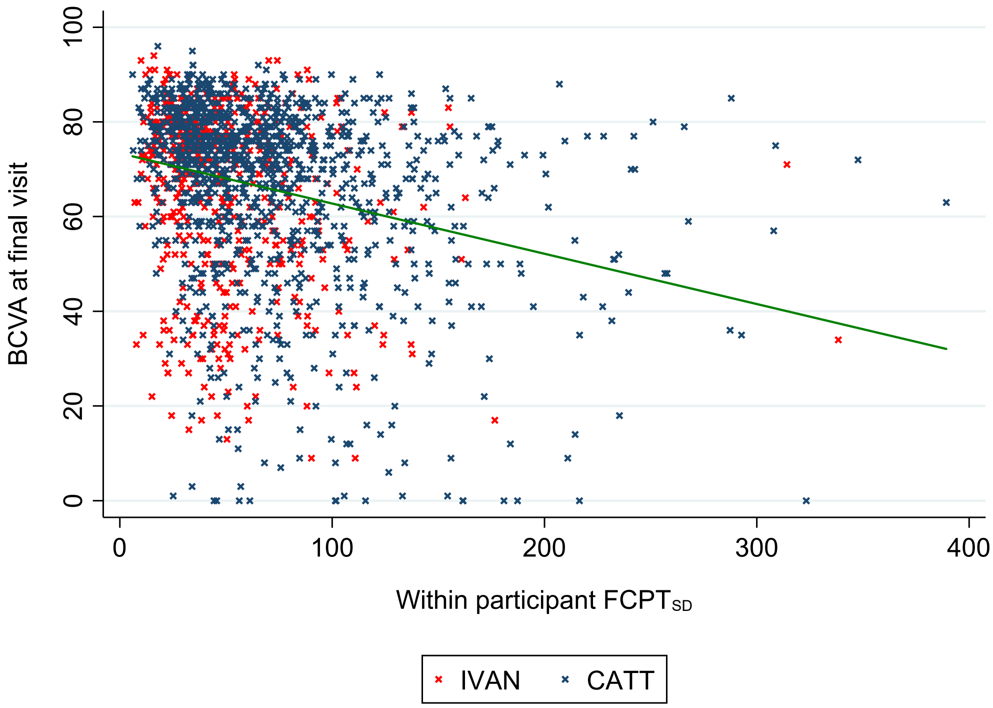

Notes: Five ETDRS letters is equivalent to one line on the ETDRS chart. Approximate Snellen equivalents for ETDRS letter scores are: 50 letters, 20/100; 55 letters, 20/80; 60 letters, 20/60; 65 letters, 20/50; 70 letters, 20/40; 75 letters, 20/30.

Abbreviations: BCVA=Best corrected visual acuity, FCPT$_{SD}$= Within participant standard deviation of foveal center point thickness

© 2020 Evans RN et al. *JAMA Ophthalmology.*

eFigure 5. Sensitivity analysis 4: estimates of associations of FCPT SD quartile with final BCVA

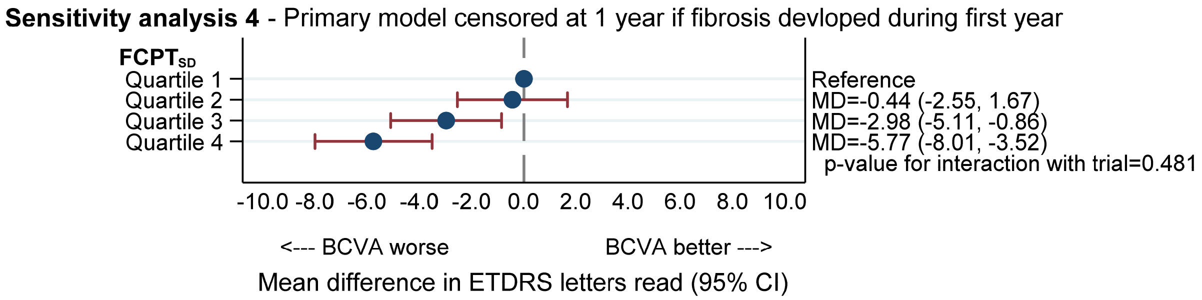

Notes: Quartile 1: $FCPT_{SD}$ < 34.01 µm. Quartile 2: $FCPT_{SD}$ ≥ 34.01 µm and <51.49 µm. Quartile 3: $FCPT_{SD}$ ≥ 51.49 µm and <80.59 µm. Quartile 4: $FCPTS_{SD}$ ≥80.59 µm

Sensitivity analysis 4 was fitted to data for 1436 participants (1731- 5[a] – 146[b] – 142[c] – 2[d])

[a] n=5 outliers removed

[b] n=146 participants with fibrosis present or data not available at baseline.

[c] n=142 participants with fibrosis data not available at final visit.

[d] n=2 participants missing final BCVA reading

Abbreviations: BCVA=Best corrected visual acuity, $FCPT_{SD}$= Within participant standard deviation of foveal center point thickness, MD=Mean difference, CI=Confidence interval, ETDRS= Early Treatment Diabetic Retinopathy Study

© 2020 Evans RN et al. *JAMA Ophthalmology.*

eFigure 6. Estimates of associations of FCPT SD quartile with final BCVA by low/high average FCPT (interaction)

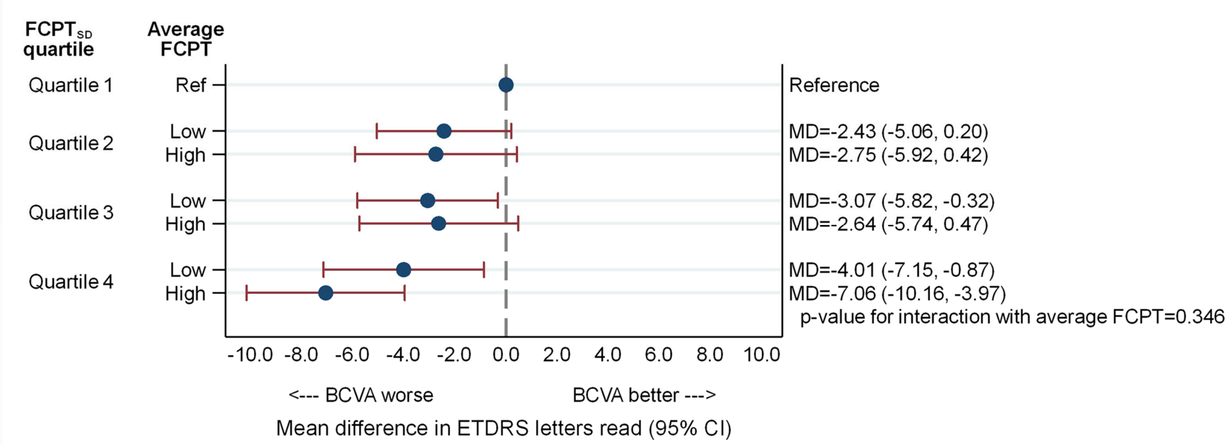

Notes:
Quartile 1: $FCPT_{SD}$ < 34.01 µm. Quartile 2: $FCPT_{SD}$ ≥ 34.01 µm and <51.49 µm. Quartile 3: $FCPT_{SD}$ ≥ 51.49 µm and <80.59 µm.
Quartile 4: $FCPT_{SD}$ ≥80.59 µm
Low average FCPT: ≤296 µm. High average FCPT: >296 µm
Model adjusted for baseline BCVA, trial, drug and dosing regimen.
Analysis fitted to n =1720 participants (1731 – 6[a] – 5[b]).
[a] n=6 participants missing final BCVA reading [b] n=5 outliers removed.

Abbreviations: BCVA=Best corrected visual acuity, $FCPT_{SD}$= Within participant standard deviation of foveal center point thickness, MD=Mean difference, CI=Confidence interval, ETDRS= Early Treatment Diabetic Retinopathy Study

© 2020 Evans RN et al. *JAMA Ophthalmology.*

eFigure 7. Estimates of associations of FCPT SD quartile with final BCVA by treatment regimen trial allocation (interaction)

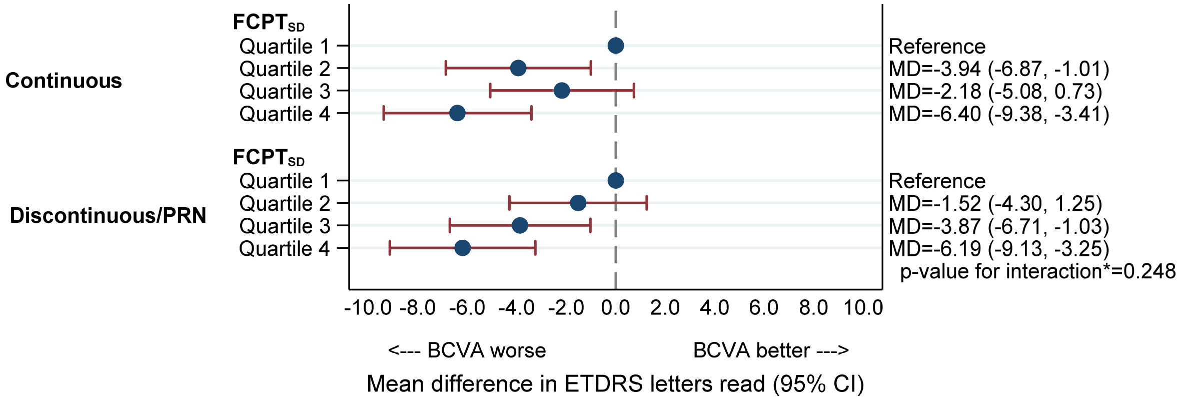

* P-value for interaction between FCPT$_{SD}$ quartile and treatment regimen trial allocation (continuous or discontinuous/PRN)

Abbreviations: BCVA=Best corrected visual acuity, FCPT$_{SD}$= Within participant standard deviation of foveal center point thickness, MD=Mean difference, CI=Confidence interval, PRN=Pro re nata, ETDRS= Early Treatment Diabetic Retinopathy Study

© 2020 Evans RN et al. *JAMA Ophthalmology.*

eTable 5. Presence of fibrosis in study eye at baseline and follow-up by FCPT SD quartile

Trial			FCPT$_{SD}$ quartile Quartile 1 (n=433)	Quartile 2 (n=433)	Quartile 3 (n=433)	Quartile 4 (n=432)	Overall (n=1731)
			n (%)	n (%)	n (%)	n (%)	n (%)
**Overall**	**Baseline**	**Yes**	45/431 (10.4%)	34/431 (7.9%)	24/430 (5.6%)	32/428 (7.5%)	135/1720 (7.8%)
		**No**	386/431 (89.6%)	397/431 (92.1%)	406/430 (94.4%)	396/428 (92.5%)	1568/1720 (92.2%)
	**Developed by final visit [a]**		186/360 (51.7%)	193/357 (54.1%)	198/360 (55.0%)	212/366 (57.9%)	789/1443 (54.7%)
**IVAN**	**Baseline**	**Yes**	37/219 (16.9%)	25/145 (17.2%)	15/120 (12.5%)	13/74 (17.6%)	90/558 (16.1%)
		**No**	182/219 (83.1%)	120/145 (82.8%)	105/120 (87.5%)	61/74 (82.4%)	468/558 (83.9%)
	**Developed by final visit [b]**		116/169 (68.6%)	84/105 (80.0%)	68/98 (69.4%)	42/55 (76.4%)	310/427 (72.6%)
**CATT**	**Baseline**	**Yes**	8/212 (3.8%)	9/286 (3.1%)	9/310 (2.9%)	19/354 (5.4%)	45/1162 (3.9%)
		**No**	204/212 (96.2%)	277/286 (96.9%)	301/310 (97.1%)	335/354 (94.6%)	1117/1162 (96.1%)
	**Developed by final visit [c]**		70/191 (36.6%)	109/252 (43.3%)	130/262 (49.6%)	170/311 (54.7%)	479/1016 (47.1%)

Notes: Denominator for development is restricted to participants who did not have fibrosis present at baseline
[a] *Data at final visit missing for 142 participants (26 Quartile 1, 40 Quartile 2, 46 Quartile 3, 40 Quartile 4)*
[b] *Data at final visit missing for 41 participants (13 Quartile 1, 15 Quartile 2, 7 Quartile 3, 6 Quartile 4)*
[c] *Data at final visit missing for 101 participants (13 Quartile 1, 25 Quartile 2, 39 Quartile 3, 24 Quartile 4)*
Abbreviations: FCPT$_{SD}$= Within participant standard deviation of foveal center point thickness

© 2020 Evans RN et al. *JAMA Ophthalmology.*

eFigure 8. Sensitivity analysis 4: estimates of associations of FCPT SD quartile with development of fibrosis

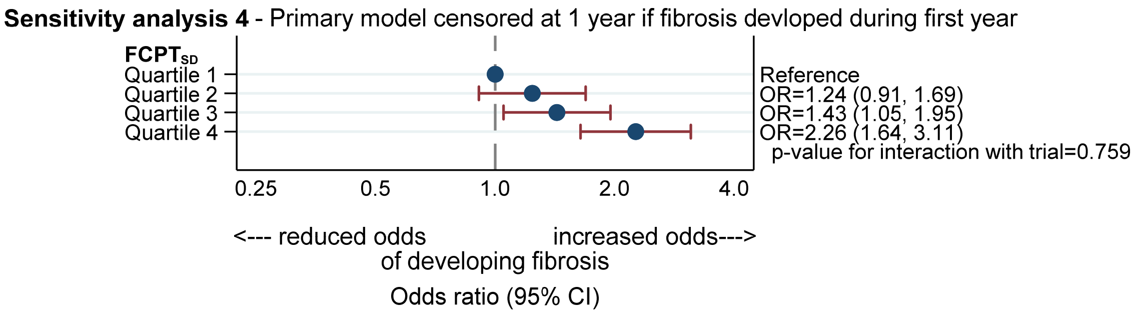

Notes: Quartile 1: $FCPT_{SD}$ < 34.01 µm. Quartile 2: $FCPT_{SD}$ ≥ 34.01 µm and <51.49 µm. Quartile 3: $FCPT_{SD}$ ≥ 51.49 µm and <80.59. Quartile 4: $FCPT_{SD}$ ≥80.59 µm

Models were restricted to participants with fibrosis absent at baseline and data available at final visit (n =1443)

Sensitivity analysis 4 was fitted to data for 1443 participants (516/789 developed fibrosis during first year and censored at 1 year)

Abbreviations: $FCPT_{SD}$= Within participant standard deviation of foveal center point thickness, OR=Odds ratio, CI=Confidence interval

© 2020 Evans RN et al. *JAMA Ophthalmology.*

eFigure 9. Sensitivity analysis 3 by trial: estimates of associations of FCPT SD quartile with development of fibrosis

Sensitivity analysis 3 - Primary model restricted to discontinuous/PRN group

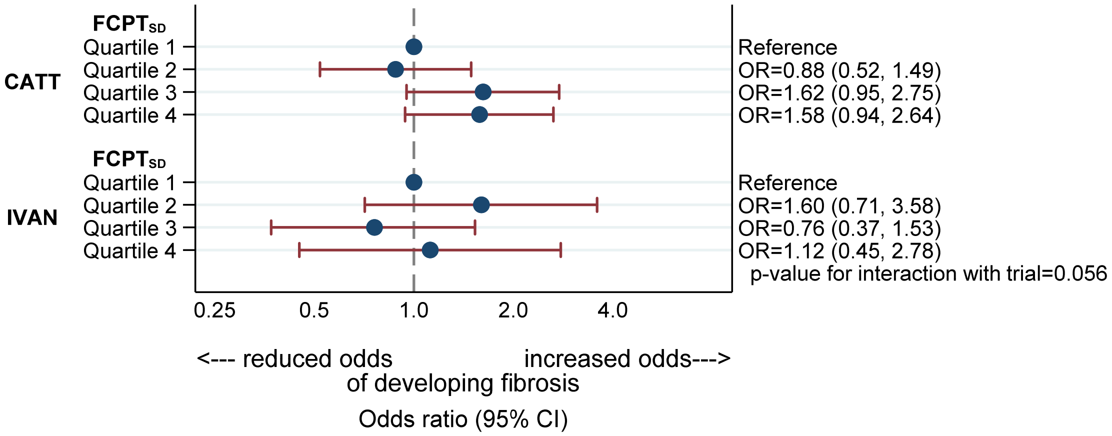

Notes: Quartile 1: $FCPT_{SD}$ < 34.01 µm. Quartile 2: $FCPT_{SD}$ ≥ 34.01 µm and <51.49 µm. Quartile 3: $FCPT_{SD}$ ≥ 51.49 µm and <80.59. Quartile 4: $FCPT_{SD}$ ≥80.59 µm
Sensitivity analysis 3 fitted to n=718 participants (502 CATT, 216 IVAN)

Abbreviations: $FCPT_{SD}$= Within participant standard deviation of foveal center point thickness, OR=Odds ratio, CI=Confidence interval, PRN=Pro re nata

© 2020 Evans RN et al. *JAMA Ophthalmology.*

eFigure 10. Estimates of associations of FCPT SD quartile with development of fibrosis by low/high average FCPT (interaction)

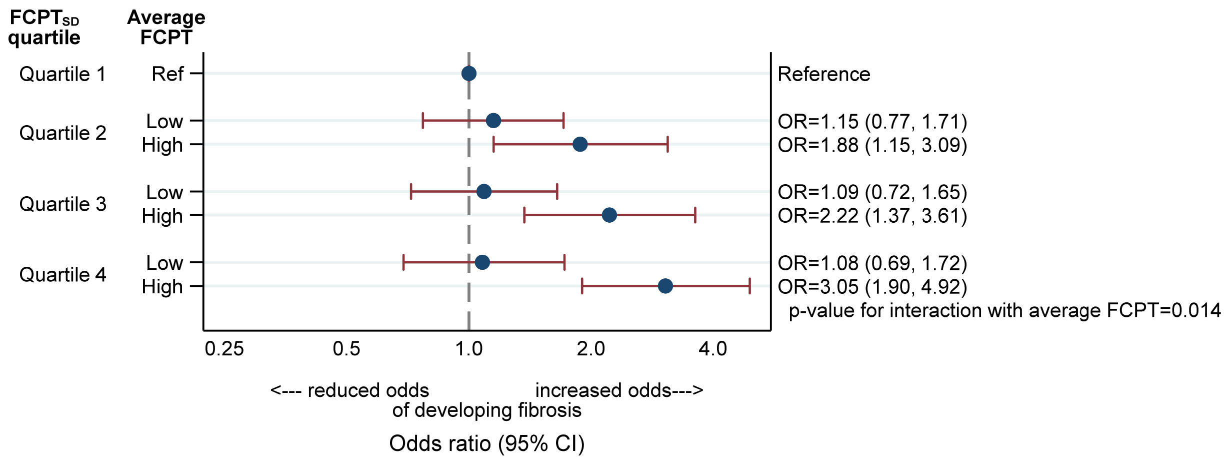

Notes:
Quartile 1: $FCPT_{SD}$ < 34.01 µm. Quartile 2: $FCPT_{SD}$ ≥ 34.01 µm and <51.49 µm. Quartile 3: $FCPT_{SD}$ ≥ 51.49 µm and <80.59.
Quartile 4: $FCPT_{SD}$ ≥80.59 µm
Low average FCPT: ≤296 µm. High average FCPT: >296 µm
Model adjusted for trial, drug and dosing regimen.
Models restricted to participants with fibrosis absent at baseline and data available at final visit (n =1443)

Abbreviations: $FCPT_{SD}$= Within participant standard deviation of foveal center point thickness, OR=Odds ratio, CI=Confidence interval, FCPT=Foveal center point thickness

© 2020 Evans RN et al. *JAMA Ophthalmology.*

eFigure 11. Estimates of associations of FCPT SD quartile with development of fibrosis by treatment regimen trial allocation (interaction)

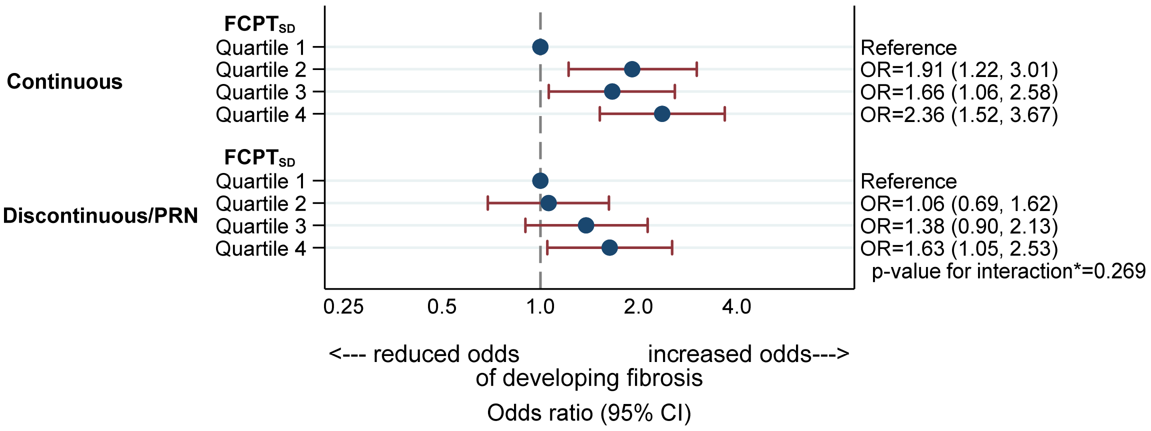

* P-value for interaction between $FCPT_{SD}$ quartile and treatment regimen trial allocation (continuous or discontinuous/PRN)

Abbreviations: $FCPT_{SD}$= Within participant standard deviation of foveal center point thickness, OR=Odds ratio, CI=Confidence interval, PRN=Pro re nata

© 2020 Evans RN et al. *JAMA Ophthalmology.*

eFigure 12. Sensitivity analysis 4: estimates of associations of FCPT SD quartile with development of geographic atrophy

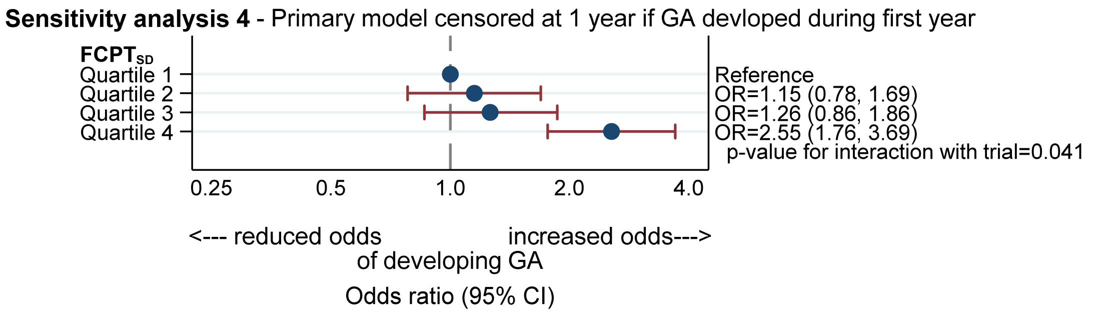

Notes: Quartile 1: $FCPT_{SD}$ < 34.01 µm. Quartile 2: $FCPT_{SD}$ ≥ 34.01 µm and <51.49 µm. Quartile 3: $FCPT_{SD}$ ≥ 51.49 µm and <80.59. Quartile 4: $FCPT_{SD}$ ≥80.59 µm
Models were restricted to participants with GA absent at baseline and data available at final visit (n =1463)
Sensitivity analysis 4 was fitted to data for 1463 participants (198/310 developed GA during first year and censored at 1 year)
Abbreviations: $FCPT_{SD}$= Within participant standard deviation of foveal center point thickness, GA=Geographic atrophy, OR=Odds ratio, CI=Confidence interval

© 2020 Evans RN et al. *JAMA Ophthalmology.*

eFigure 13. Estimates of associations of FCPT SD quartile with development of geographic atrophy by treatment frequency allocation (interaction)

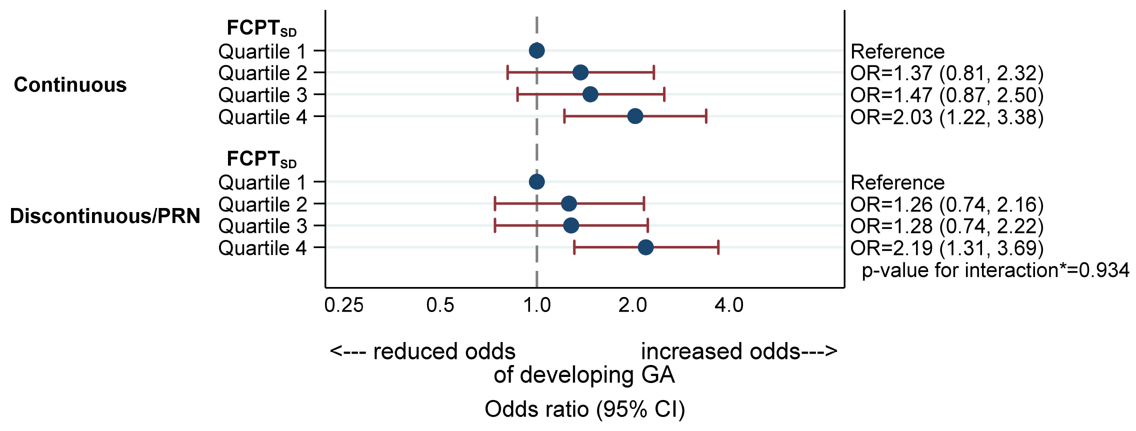

* P-value for interaction between FCPT$_{SD}$ quartile and treatment regimen trial allocation (continuous or discontinuous/PRN)

Abbreviations: FCPT$_{SD}$= Within participant standard deviation of foveal center point thickness, GA=Geographic atrophy. OR=Odds ratio, CI=Confidence interval, PRN=Pro re nata

© 2020 Evans RN et al. *JAMA Ophthalmology.*

eTable 6. Post hoc analysis: association of total number of injections with FCPT SD (discontinuous/PRN group only)

**Model**	**OR (95% CI)**	**p-value**
Number of injections during trial (per 3 injections)		
Model 1: Adjusted for trial and drug allocation	1.13 (1.07, 1.20)	<0.001
Model 2: Adjusted for trial, drug allocation baseline lesion size	1.10 (1.04, 1.17)	0.001

Notes: Linear regression could not be used to assess the effect of the number of injections on continuous $FCPT_{SD}$ as the assumptions of linear regression were not met. Therefore, $FCPT_{SD}$ was analyzed using ordinal logistic regression based on quartiles of $FCPT_{SD}$.
Model restricted to discontinuous/PRN group only (n=877)
Models 1 fitted to n=877 participants
Model2 fitted to n =840 participants (877 – 37 [a])
[a] n=37 participants with data missing for baseline lesion size

Abbreviations: $FCPT_{SD}$= Within participant standard deviation of foveal center point thickness, OR=Odds ratio, CI=Confidence interval

© 2020 Evans RN et al. *JAMA Ophthalmology.*
